# Random and Diblock Thermoresponsive Oligo(ethylene glycol)-Based Copolymers Synthesized via Photo-Induced RAFT Polymerization

**DOI:** 10.3390/polym14010137

**Published:** 2021-12-30

**Authors:** Alexey Sivokhin, Dmitry Orekhov, Oleg Kazantsev, Olga Sivokhina, Sergey Orekhov, Denis Kamorin, Ksenia Otopkova, Michael Smirnov, Rostislav Karpov

**Affiliations:** 1 Laboratory of Acrylic Monomers and Polymers, Department of Chemical and Food Technologies, Dzerzhinsk Polytechnic Institute, Nizhny Novgorod State Technical University n.a. R.E. Alekseev, 24 Minin Street, 603950 Nizhny Novgorod, Russia; mitriy07@mail.ru (D.O.); kazantsev@dpingtu.ru (O.K.); orekhov807@gmail.com (S.O.); d.kamorin@mail.ru (D.K.); k.otopkova@gmail.com (K.O.); thelordoftime@yandex.ru (M.S.); r_karpov@mail.ru (R.K.); 2 V.A. Kargin Research Institute of Chemistry and Technology of Polymers with Pilot Plant, 606000 Dzerzhinsk, Russia; olgasivokhina@yandex.ru; 3 Chromatography Laboratory, Department of Production Control and Chromatography Methods, Lobachevsky State University of Nizhni Novgorod, Dzerzhinsk Branch, 23 Prospekt Gagarina, 603950 Nizhny Novgorod, Russia

**Keywords:** photoiniferter polymerization, thermoresponsive polymer, block copolymer, self-assembly, self-folding, oligo(ethylene glycol) methacrylates

## Abstract

Amphiphilic random and diblock thermoresponsive oligo(ethylene glycol)-based (co)polymers were synthesized via photoiniferter polymerization under visible light using trithiocarbonate as a chain transfer agent. The effect of solvent, light intensity and wavelength on the rate of the process was investigated. It was shown that blue and green LED light could initiate RAFT polymerization of macromonomers without an exogenous initiator at room temperature, giving bottlebrush polymers with low dispersity at sufficiently high conversions achieved in 1–2 h. The pseudo-living mechanism of polymerization and high chain-end fidelity were confirmed by successful chain extension. Thermoresponsive properties of the copolymers in aqueous solutions were studied via turbidimetry and laser light scattering. Random copolymers of methoxy- and alkoxy oligo(ethylene glycol) methacrylates of a specified length formed unimolecular micelles in water with a hydrophobic core consisting of a polymer backbone and alkyl groups and a hydrophilic oligo(ethylene glycol) shell. In contrast, the diblock copolymer formed huge multimolecular micelles.

## 1. Introduction

In the last decade, methods of controlled photopolymerization induced by visible light have attracted great interest. The simplicity of the experimental setup, lack of high temperatures, and cheap household light source coupled with good control of the reaction made this process quite popular among researchers. The simple “on/off” button control of the process makes it convenient to produce block copolymers, and the development of oxygen-insensitive polymerization methods [1,2,3,4] can be useful in producing various coatings. The low-temperature process without a thermal initiator allows the use of water as a green solvent in preparing thermoresponsive polymers at temperatures below LCST [5]. Moreover, an essential advantage of photoRAFT polymerization is high chain-end fidelity [6,7], making it one of the best tools for precision polymer synthesis. The use of continuous flow reactors for photoRAFT polymerization, in addition to the mentioned advantages, also makes it possible to significantly reduce reaction times and achieve high conversions while maintaining good control over the molecular weight distribution [8].

Three basic mechanisms of photoRAFT polymerization are distinguished: (1) photoiniferter polymerization [9,10,11], (2) RAFT polymerization with a photoinitiator [7,12,13], and (3) photo-induced electron/energy transfer RAFT (PET-RAFT) polymerization [1,10,14]. In the first case, radicals are formed by direct photolytic cleavage of the chain transfer agent (CTA); in the second case, they are produced from the photoinitiator when irradiated with visible light. The third method is based on the use of photoredox catalysts, which reduce CTA when exposed to light, yielding free radicals. The photophysical aspects of initiation via several common CTAs were qualitatively investigated in [10], and the first attempts at kinetic analysis of the process were made in [14].

Most studies are focused on the typical monomers involved in radical polymerization: methyl methacrylate, methyl acrylate, dimethylacrylamide, styrene, etc. [12,15,16,17,18,19,20,21,22]. Far less research has been conducted on the preparation of bottlebrush polymers through visible light-mediated polymerization. For example, the possibility of obtaining bottlebrush polymers by the grafting-through and grafting-from strategies has been shown [3,7,9]. One-pot and one-pass photoselective processes without intermediate isolation were used to synthesize graft and branched copolymers. For example, the sequential carrying out of the processes of backbone formation with green light photoRAFT and subsequent side-chain extension with red or blue light allowed independent control over these two steps in graft copolymerization [23] and synthesis of bottlebrush polymers [11].

Solvent photoRAFT polymerization was used to obtain macroCTA, which then initiated the emulsion polymerization of styrene, acting simultaneously as a surfactant [24]. The advantage of this approach was that resulting micellar nanoobjects could be tuned in size by controlling the DP of the second block. Molecularly imprinted polymers specific for testosterone, a model template, were obtained using blue (435 nm) or green (525 nm) light irradiation [25].

In recent years, biocompatible polymers of PEGMA and its hydrophobically modified copolymers have shown interesting properties: in particular, the ability to form single chain nanoparticles (SCNPs) in aqueous solutions [26,27,28,29,30,31], which may become promising polymeric nanocontainers for hydrophobic drug delivery. The aim of this work was to investigate the synthesis of PEG-based bottlebrushes (homopolymers, random and diblock copolymers) using visible light-mediated RAFT polymerization.

## 2. Materials and Methods

### 2.1. Materials

**Figure 1:**
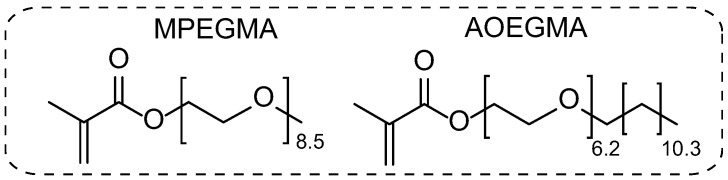
Monomer structures and designations.

Methoxy oligo(ethylene glycol)_8.5_ methacrylate (MPEGMA, Mn = 500) from Sigma-Aldrich (Saint Louis, MO, USA) and alkoxy(C_12_–C_14_) oligo(ethylene glycol)_7.2_ methacrylate (AOEGMA) (Figure 1) synthesized according to Section 2.2 were purified from the inhibitor by passing through a column filled with basic alumina. Chain transfer agent 4-cyano-4-[(dodecylsulfanylthiocarbonyl)sulfanyl]pentanoic acid (CDTPA) was synthesized according to the procedure in [32]. All solvents (toluene, dimethyl sulfoxide, tetrahydrofuran (ACS reagent, ≥99.5%), methylene chloride, acetonitrile (for spectroscopy, ≥99.5%), and hexane (Laboratory Reagent, ≥95%) from Sigma-Aldrich (Saint Louis, MO, USA) were used without purification.

### 2.2. Synthesis of AOEGMA

AOEGMA was synthesized by the esterification of methacrylic acid (MA) with a mixture of industrial ethoxylated higher fatty alcohols of C_12_–C_14_ fraction (weight ratio C_12_/C_14_ of 3.4:1) from the “Sintanol Plant” (Dzerzhinsk, Russia) at a temperature of 120 °C in a toluene solution (toluene content of 30 wt%) in the presence of 2 wt% of p-toluene sulfonic acid as a catalyst and 0.3 wt% of hydroquinone as a polymerization inhibitor. The initial reagents (MA to alcohol) ratio was 3.0:1.0 (mol.). The resulting reaction mixture was diluted with 10-fold chloroform and washed several times with 5% alkali solution to remove MA and a major amount of hydroquinone. After washing, the solvent was removed at reduced pressure using a rotary evaporator. The monomer yield was determined gravimetrically and was equal to 85%. Monomer purity (98.6%) was determined by the content of C=C double bonds using bromide–bromate titration. ^1^H NMR [400 MHz, chloroform-d, 25 °C, δ = 7.27 (chloroform)]: δ 6.11 (CH_2_=), 5.55 (CH_2_=), 4.28 (COOCH_2_-), 3.71–3.42 (-CH_2_O(CH_2_CH_2_O)_n_CH_2_-), 1.93 (CH_2_=C(CH_3_)COO-), 1.55 (-OCH_2_CH_2_(CH_2_)_m_CH_3_), 1.24 (-OCH_2_CH_2_(CH_2_)mCH_3_), 0.86 (-OCH_2_CH_2_(CH_2_)_m_CH_3_).

### 2.3. Photoiniferter RAFT Polymerization

**Figure 2:**
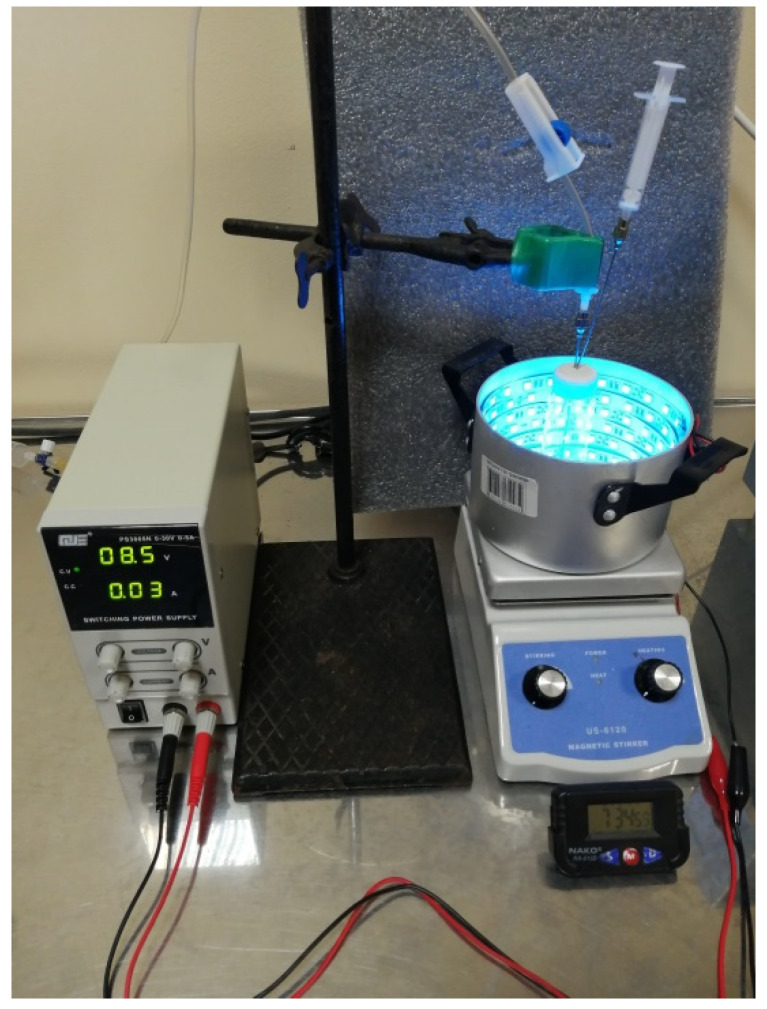
Experimental set-up for the photoiniferter polymerization.

Polymerizations were conducted in 4–20 mL screw-capped vials (Macherey-Nagel). A photoreactor was an aluminum cylinder 12 cm in diameter and 8 cm high, with an LED strip stuck on the inner side (Figure 2).

The light sources were 5050 SMD LEDs (Wenzhou Rockgrand Trade Co., Ltd., Wenzhou, China, 60 LEDs per meter, maximum power of 14.4 W/m at 12 V), set to green (λmax = 520 nm) and blue (λmax = 470 nm). The light intensity was adjusted with a PS3005N switching power supply from QJE (Xinyujie Electronics Co., Ltd., Shenzhen, China) and measured with an OHSP-350C Spectral Analyzer (Hangzhou Hopoo Light&Color Technology Co., Ltd., Hangzhou, China).

A typical RAFT photopolymerization procedure was as follows: CDTPA (5.5 mg, 13.2 μmol, 1.0 eq) and MPEGMA (1.25 g, 2.65 mmol, 200 eq) were dissolved in THF (1.25 g), stirred (ca. 600 rpm) until completely dissolved and placed in a photoreactor. The total concentration of the monomers and CTA was kept at 50%. The reaction mixture was purged with N_2_ for 15 min, and polymerization was initiated by irradiation with corresponding LEDs (7.0–8.3 mW/cm^2^). During the polymerization, samples of the reaction mixture were taken with a syringe in a nitrogen atmosphere to avoid contact with oxygen and diluted with acetonitrile to determine monomer conversions by HPLC.

The polymerizations were quenched by exposing the mixtures to air and cooling in the dark, followed by precipitation with an appropriate nonsolvent. The resulting homopolymers and random copolymers were purified via multiple precipitations from toluene or THF solutions using hexane (for pMPEGMAs) or acetonitrile (for pAOEGMAs), followed by vacuum drying. Random copolymers synthesized in DMSO, as well as block copolymers, were diluted with a tenfold volume of ethyl alcohol containing 0.05% hydroquinone and purified by dialysis (MWCO 8–14 k) against ethyl alcohol for three days in the dark and then dried in a vacuum. The compositions of copolymers were determined by HPLC based on residual monomer concentrations and ^1^H NMR. A typical polymer spectrum: ^1^H NMR [400 MHz, chloroform-d, 25 °C, δ = 7.27 (chloroform)]: δ, 4.06 (COOCH_2_-), 3.71–3.42 (-CH_2_O(CH_2_CH_2_O)_n_CH_2_-, -CH_2_O(CH_2_CH_2_O)_n_CH_3_), 3.35 (-CH_2_O(CH_2_CH_2_O)_n_CH_3_), 2.1–1.65 (-CH_2_C(CH_3_)-), 1.55 (-OCH_2_CH_2_(CH_2_)_m_CH_3_), 1.24 (-OCH_2_CH_2_(CH_2_)_m_CH3), 1.0–0.86 ((-CH_2_C(CH_3_)-, -OCH_2_CH_2_(CH_2_)_m_CH_3_).

### 2.4. Characterization Techniques

#### 2.4.1. General Methods

^1^H NMR spectra were recorded at 25 °C in CDCl_3_ or DMSO-d_6_ on an Agilent 400 MHz DD2 spectrometer. The values of dn/dc for copolymers were determined using a BI-DNDC differential refractometer (Brookhaven Instr. Corp., Holtsville, NY, USA) at 30 °C in the concentration range of 1–15 mg/mL. The concentrations of monomers in reaction mixtures were measured by HPLC using a Shimadzu Prominence chromatographic system equipped with refractometric and matrix UV detectors, a thermostat and a Kromasil 100–5-C18 4.6 × 250 mm column. Acetonitrile was used as an eluent, the flow rate was 0.9 mL/min, and the thermostat temperature was 55 °C.

Molecular weights and molecular weight distributions of polymers were determined by GPC using a Chromos LC-301 instrument with an Alpha-10 isocratic pump, a Waters 410 refractometric detector and two exclusive columns, Phenogel 5 µm 500A and Phenogel 5 µm 10E5A, from Phenomenex (with a measurement range from 1 k to 1000 k); tetrahydrofuran was used as an eluent. Polystyrene standards were used for calibration.

Differential scanning calorimetry (DSC) was performed for polymer samples (ca. 10–15 mg in an aluminum pan) under dry argon flow on a DSC 204F1 Phoenix calorimeter (Netzsch, Selb, Germany) equipped with CC 200 controller for liquid nitrogen cooling. The heating and cooling rates were 10 °C/min and −10 °C/min, respectively, between −80 °C and 80 °C.

#### 2.4.2. Dynamic (DLS) and Static (SLS) Light Scattering

Laser light scattering (LLS) experiments were performed using a Photocor Complex multi-angle light scattering instrument (Photocor Ltd., Moscow, Russia) equipped with a thermostabilized diode laser (λ = 659 nm, 35 mW) and a thermo-electric Peltier temperature controller (temperature range from 5 to 100 °C, accuracy of 0.1 °C). LLS was used to determine hydrodynamic radii (Rh) of polymer molecules and micelles (DLS), weight average molecular weights (M_W_), second virial coefficients (A_2_), and aggregation numbers (N_agg_) of micelles (SLS).

After preparation, polymer solutions were kept at room temperature for 24 h to reach equilibrium and were filtered through CHROMAFIL PET syringe filters (0.20 μm) before starting measurements. At least three measurements were taken for each sample, resulting in an average hydrodynamic radius Rh (nm). M_W_ and A_2_ were determined using the single-angle Debye plot method.

The scattering geometry of the instrument used was as follows: a vertically polarized incident light and detection without a polarizer (VU geometry, Rv). The Rayleigh ratio for toluene at the incident wavelength of 659 nm and measurement temperature was calculated according to [33].

#### 2.4.3. Turbidimetry

Turbidimetry was used to determine solution cloud points (C_p_), i.e., phase transition temperatures. Aqueous polymer solutions with concentrations of 1% (wt.) were used for the experiments; the rate of heating was approximately 0.3 °C/min. The C_p_ values were determined as a position of the maximum of the first derivative of the s-shaped turbidity curve [34]. Optical transmittance was measured using a KFC-2MP colorimeter (Zagorsk Optical and Mechanical Plant, Sergiev Posad, Russia) at a wavelength of 540 nm.

## 3. Results and Discussion

### 3.1. Photoiniferter RAFT Homopolymerization in Different Solvents

**Figure 3:**
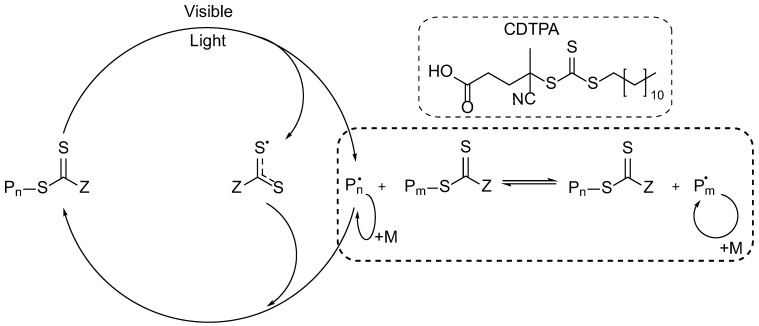
Mechanism of photoiniferter polymerization under visible light.

**Figure 4:**
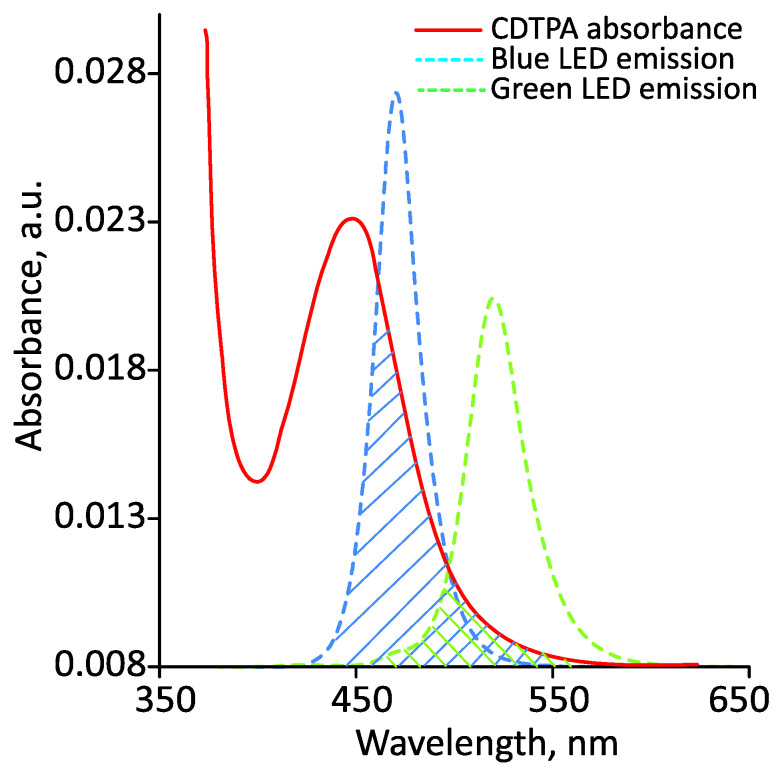
Absorption spectrum of photoiniferter (CDTPA) measured using UV–Vis spectroscopy, and emission spectra of green and blue LEDs. The peaks of the emission spectra were at 470 and 520 nm for blue and green LEDs, respectively.

Photoiniferter RAFT polymerization assumes the use of light for the direct photolytic cleavage of a CTA followed by the switching of the process to the RAFT mode (Figure 3). 4-cyano-4-[(dodecylsulfanylthiocarbonyl)sulfanyl]pentanoic acid (CDTPA) was used as a CTA. Green and blue LEDs with wavelengths λmax = 520 nm and 470 nm, respectively, were used as light sources in this work. The UV–visible spectrum of CDTPA in acetonitrile is shown in Figure 4. It has a maximum absorbance of approximately 450 nm corresponding to the forbidden n to π* electronic transition [35]. The CDTPA absorbance peak overlaps the emission spectra of green and blue LEDs (Figure 4), which enables its direct photolysis under visible light.

**Figure 5:**
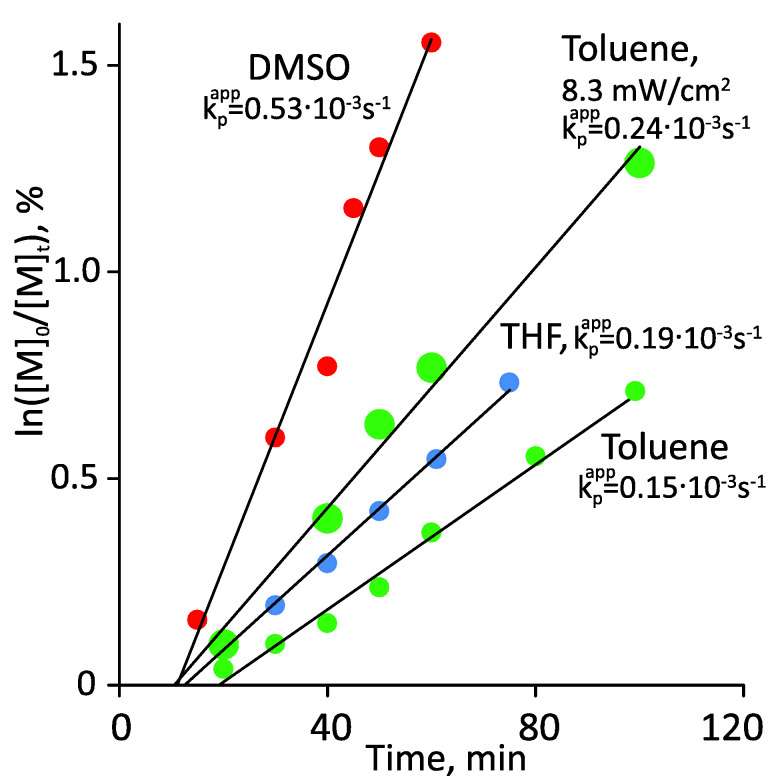
Kinetic first-order plot for the photo-induced RAFT polymerization of MPEGMA without an initiator in different solvents. Polymerization conditions: ω_MPEGMA_ = 50 wt.%, [MPEGMA]_0_/[CDTPA]_0_ = 200:1, blue LED, 7 mW/cm^2^ (unless otherwise specified), 35 °C.

The study started with the search for optimal polymerization conditions: solvent, wavelength, and intensity of radiation, as well as the presence of agitation. Toluene, DMSO, and THF were tested as solvents. In terms of convenience of carrying out the process and subsequent isolation of polymers, toluene is the preferred solvent, as it has lower volatility compared to THF and allows easy polymer isolation by precipitation with acetonitrile and hexane for p(AOEGMA) and p(MPEGMA), respectively. However, obtaining the polymers in toluene proved to be a non-trivial task. Polymerization began only after careful removal of oxygen from the reaction mixture and if sampled in a nitrogen atmosphere, and exhibited the lowest rate among all tested solvents (Figure 5). DMSO showed the highest rate of the process and the smallest induction period, which was due to low oxygen solubility and its ability to bind oxygen through forming dimethyl sulfone [4,36]. In DMSO, the induction period usually did not exceed 10 min, whereas in toluene, under green light, it could reach several hours. However, the isolation of polymers from DMSO through precipitation was difficult; effective purification from residual monomers could be achieved only by dialysis or preparative chromatography. THF ranked between toluene and DMSO in terms of polymerization rate.

Regarding stirring, there was no difference in the reaction rate with and without stirring. Figure 5 shows that all polymerizations obeyed pseudo-first-order kinetics within 120 min. When the irradiation intensity increased from 7.0 to 8.3 mW/cm_2_, the polymerization rate increased more than 1.5-fold. This is clearly seen from the comparison of the apparent propagation rate constants calculated as the slopes of the kinetic dependences in the ln([M]_0_/[M]_t_)–time coordinates.

**Figure 6:**
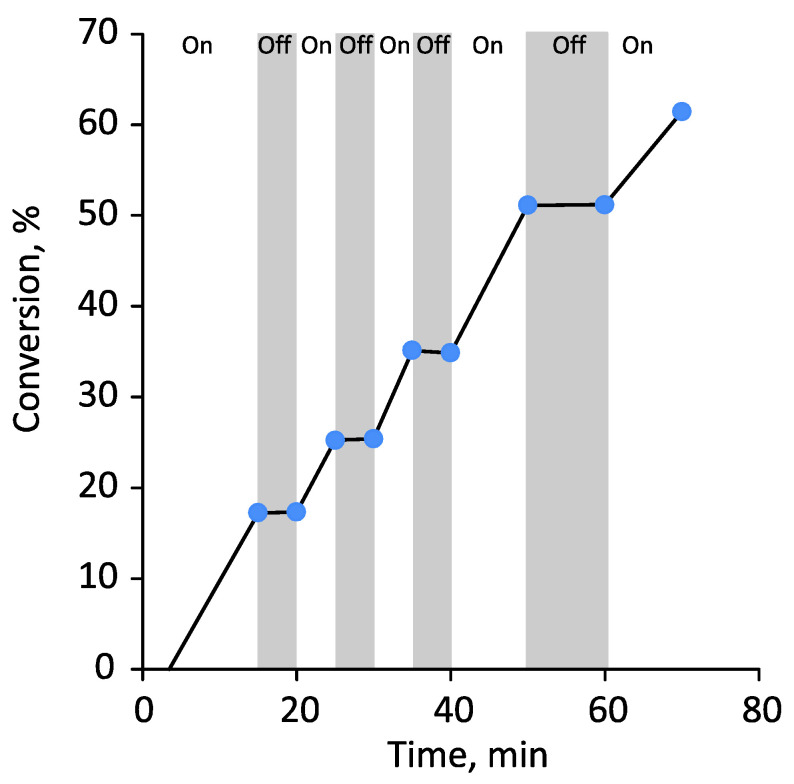
“On/off” experiment for the photo-induced RAFT polymerization of MPEGMA in DMSO. Polymerization conditions: ω_MPEGMA_ = 50 wt.%, [MPEGMA]_0_/[CDTPA]_0_ = 200:1, blue LED, 7 mW/cm^2^, 35 °C.

To demonstrate the pseudo-living nature of the polymerization and the easy switchability of the process, an “on-off” experiment was performed. Figure 6 demonstrates that polymerization was completely stopped (according to HPLC) during the dark period and easily re-initiated again when the light was turned on, proceeding at approximately the same rate.

The results of all the polymerizations performed are summarized in Table 1. As can be seen, the process was characterized by fairly good control of the MWD with a dispersity mainly in the range 1.2–1.3. The exceptions were the experiments carried out in green light; in this case, apparently, the duration of the process had a decisive influence on dispersity. The reaction rate in green light was much lower. This is due to the different absorption intensities of CDTPA within the visible-light spectrum of blue and green LEDs (Figure 4, note the overlapping areas), so that the polymerization rates show a wavelength dependence.

Noteworthy is the poor agreement between the theoretical molecular weights and the GPC data, which were severely underestimated. This has been repeatedly reported for bottlebrushes based on oligo(ethylene glycol)-containing macromonomers [30,37,38,39,40]. As shown below, the main reason was the nonlinear dependence of the retention time on the molecular weight in GPC, which had a maximum; researchers who were working in the low-molecular-weight region did not note this feature [3].

### 3.2. Photoiniferter RAFT Copolymerization

**Figure 7:**
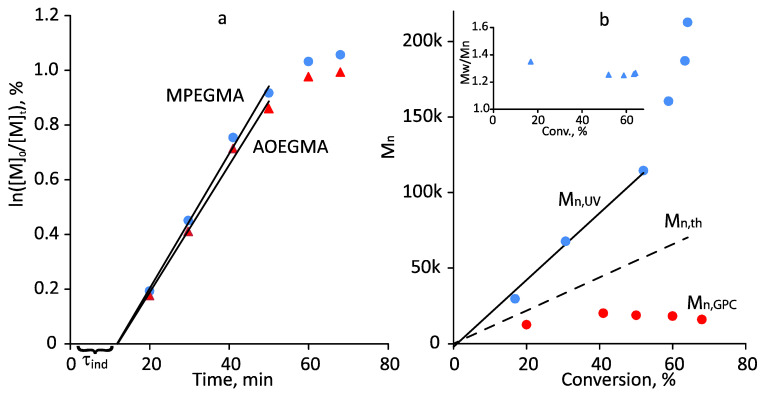
Kinetic first-order plot (**a**), and the plot of M_n,UV_, M_n,th_, M_n,GPC_ and M_W_/M_n_ vs. conversion (**b**) for the photo-induced RAFT copolymerization of MPEGMA and AOEGMA in toluene (Ph11). Polymerization conditions: ω_MPEGMA_ = 50 wt.%, [MPEGMA]_0_/[AOEGMA]_0_/[CDTPA]_0_ = 100:100:1, blue LED, 8.3 mW/cm^2^, 35 °C.

**Figure 8:**
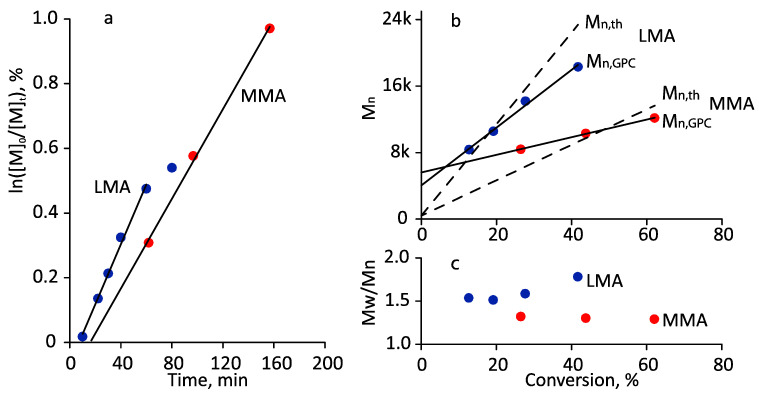
Kinetic first-order plot (**a**), and the plot of M_n,th_, M_n,GPC_ (**b**) and M_W_/M_n_ (**c**) vs. conversion for the photo-induced RAFT polymerization of MMA and LMA in DMSO. Polymerization conditions: ω_monomer_ = 50 wt.%, [Monomer]_0_/[CDTPA]_0_ = 200:1, green LED, 7.3 mW/cm^2^, 35 °C.

Figure 7 represents the kinetics of MPEGMA–AOEGMA (1:1) copolymerization and the dependence of the number average molecular weight of the copolymers on the conversion. A few earlier experiments were confusing: the molecular weight (GPC) decreased with conversion, giving the impression that the process proceeded not in a pseudo-living mode but a depolymerization mode, although the kinetic curves (Figure 7a) indicated the opposite: linearity was observed until the conversion of 50%. Similar phenomena were observed in [41], to a greater extent for linear polymers than for branched polymers. Experiments performed under similar conditions using monomers such as methyl methacrylate (MMA) and lauryl methacrylate (LMA) confirmed the pseudo-living nature of the polymerization: M_n_ increased linearly with conversion (Figure 8).

Thanks to the extensive work done by Skrabania et al. [42] on studying the absorption characteristics of a large set of thiocarbonyl CTAs in different solvents, the determination of number average molecular weights for OEGMA-based polymers is not difficult while being characterized by fairly high accuracy.

**Figure 9:**
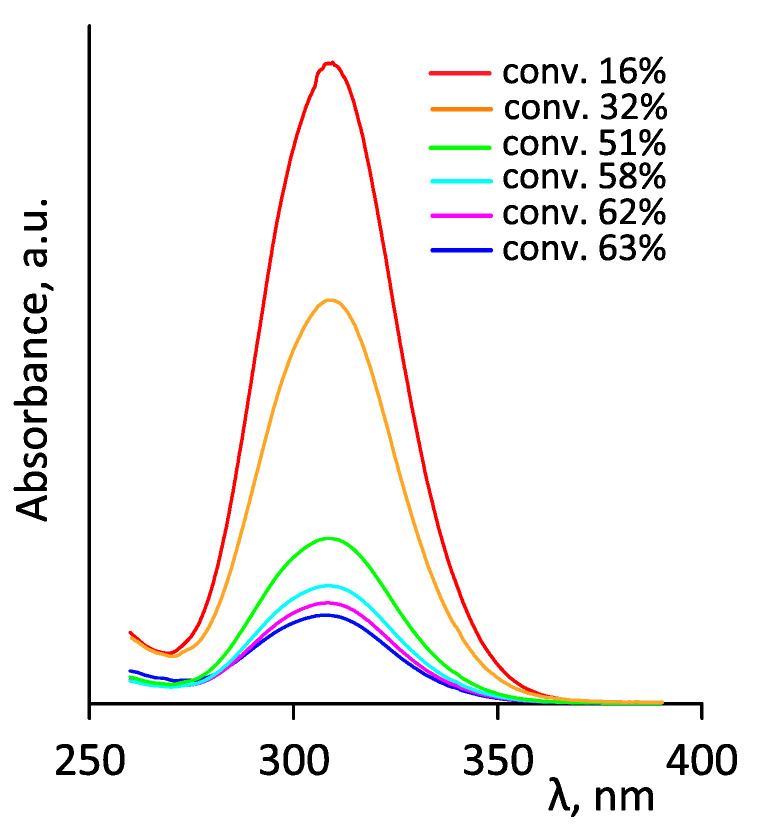
UV–vis spectra of MPEGMA–AOEGMA copolymers in acetonitrile (Ph11 series). Maximum absorption wavelength λmax = 307 nm.

Figure 9 shows the spectra of MPEGMA–AOEGMA copolymers isolated at different conversions; the corresponding M_n,UV_ values calculated according to [42] are shown in Figure 7b. A linear dependence up to a conversion of ~55% was also observed in this case. The higher M_n,UV_ values compared to M_n,th_ can be explained by the presence of small amounts of dimethacrylates (formed during macromonomer production and which are difficult to remove) leading to rare cross-links. At high conversions, irreversible chain termination reactions could obviously take place.

**Figure 10:**
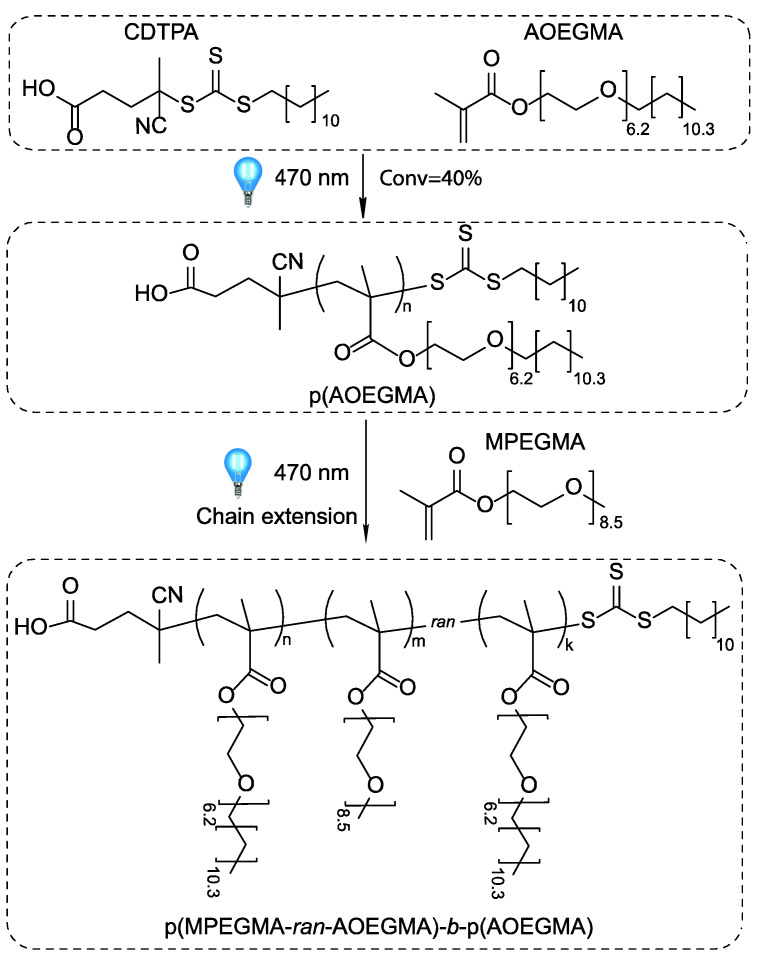
Photo-induced RAFT copolymerization of MPEGMA and AOEGMA for the synthesis of a diblock copolymer under blue light irradiation in THF.

To evaluate the fidelity of the RAFT end group, a chain extension experiment from Ph13 was performed with two monomers to obtain a water-soluble micelle-forming copolymer. The scheme of block copolymerization is shown in Figure 10.

Under blue light (8.3 mW/cm^2^), the first AOEGMA block reached a monomer conversion of 40% after 60 min, with M_n,UV_ = 112,600 g/mol and a narrow molecular weight distribution (PDI = 1.19). Successive chain extension with MPEGMA and AOEGMA (62.5:37.5 mol) reached monomer conversions of 44 and 40%, respectively, after 90 min (M_n,UV_ = 480,900 g/mol, PDI = 1.23, M_n,th_ = 390,000).

**Figure 11:**
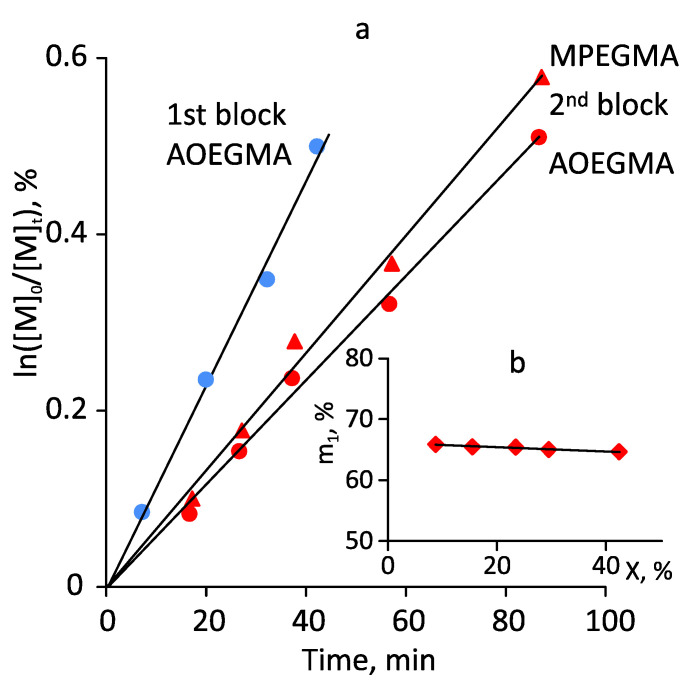
(**a**) Kinetic first-order plot for the photo-induced RAFT homopolymerization of AOEGMA (blue) and subsequent chain extension (red) with MPEGMA and AOEGMA in THF. (**b**) The plot of the 2nd block composition, m_1_, vs. conversion, X. m_1_, is the mole fraction of MPEGMA in a random 2nd block. Polymerization conditions for the first stage: ω_AOEGMA_ = 50 wt.%, [AOEGMA]_0_/[CDTPA]_0_ = 200:1, blue LED, 8.3 mW/cm^2^, 35 °C; second stage: ω_monomers_ = 49 wt.%, [MPEGMA]_0_/[AOEGMA]_0_/[macroRAFT]_0_ ≊ 800:480:1, blue LED, 8.3 mW/cm^2^, 35 °C.

Figure 11a shows data on the kinetics of monomer consumption during the obtaining of the first and second blocks. All polymerizations followed pseudo-first-order kinetics in the range of conversions investigated. The composition of the second random copolymer block according to HPLC data (based on monomer consumption) was MPEGMA/AOEGMA = 64.6:35.4 mol. (Figure 11b), while the overall block copolymer composition was 42:58 mol. according to ^1^H NMR.

**Figure 12:**
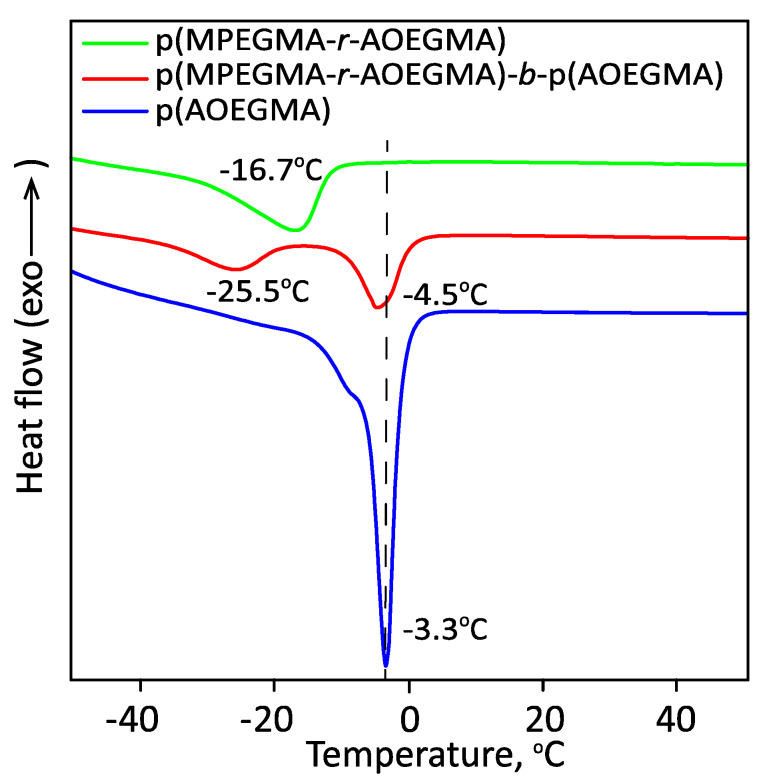
DSC thermograms of p(AOEGMA) (Ph13), p(MPEGMA-*ran*-AOEGMA) (Ph12), p(MPEGMA-*ran*-AOEGMA)-*b*-p(AOEGMA) (Ph14). Heating rate: 10 °C/min.

A good confirmation of the formation of sufficiently long blocks is their independent thermal behavior. The thermal behavior of the Ph14 block copolymer was studied using differential scanning calorimetry (DSC) in the range of −50 to 50 °C. As shown in Figure 12, the DSC curve for the AOEGMA homopolymer, Ph13, had one quite narrow peak corresponding to the melting point (−3.3 °C). The random MPEGMA/AOEGMA copolymer (56:44), Ph12, also had a single but strongly broadened peak (−16.7 °C) due to the presence of hard-to-crystallize MPEGMA units. The block copolymer, Ph14, exhibited two well-defined melting peaks indicating microphase separation. The positions of the peaks were close to those of the AOEGMA homopolymer and random copolymer, and the shift of the peak corresponding to the second copolymer block to the low-temperature region was associated with its composition, which was enriched with MPEGMA units (64.6:35.4).

### 3.3. Thermoresponsive Properties, Hydrodynamic and Molecular Weight Characteristics of the Synthesized (Co)Polymers

**Figure 13:**
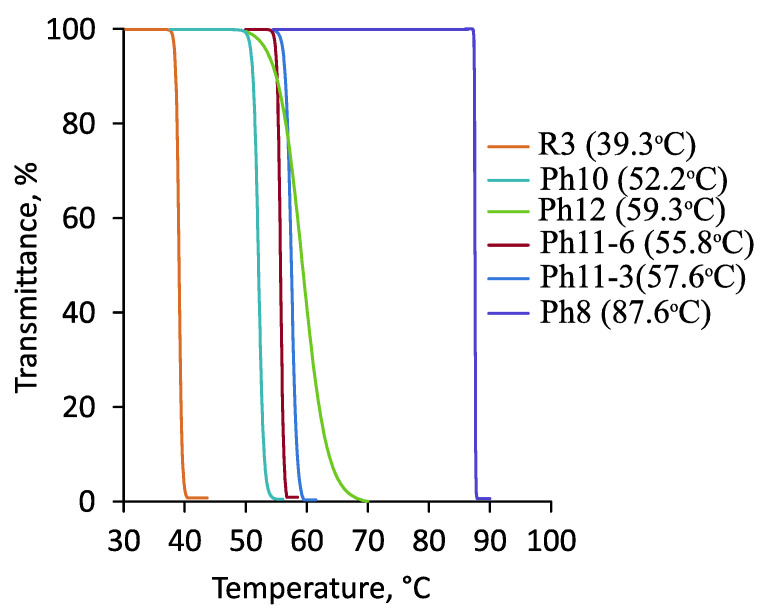
Light transmittance vs. temperature (heating cycle) for 1% aqueous solutions of samples Ph8, Ph10–Ph12 and R3.

Thermoresponsive properties of the (co)polymers were studied using turbidimetry. The dependences of light transmission on temperature were obtained for 1% aqueous solutions under a heating regime. Examples of the light transmission curves are shown in Figure 13.

The data obtained were typical for thermoresponsive amphiphilic copolymers: as the fraction of hydrophobic units increased, the cloud point (C_p_) decreased dramatically. Molecular weight had less influence on C_p_ than composition, except for low-molecular-weight polymers, in which the RAFT agent dodecyl group had a significant effect on the hydrophobic–hydrophilic balance of a macromolecule.

The MPEGMA homopolymer had the highest C_p_ in the series of samples studied. For the samples Ph11-3 and Ph11-6, with the same compositions and molecular weights M_n,UV_ equal to 114,500 and 212,700, the change in C_p_ was quite noticeable at 1.9 °C. The Ph12 sample, containing more MPEGMA units, had the highest C_p_ among the copolymers. In order to evaluate the effect of side-chain structure, data are also presented for sample R3 obtained earlier [31], which had a composition similar to Ph10 and Ph11 but differed in the number of oxyethyl fragments in MPEGMA units (7.2 instead of 8.5, see Figure 1). As can be seen from Figure 13 (compare the samples R3 and Ph11-6), adding a bit more than one oxyethyl fragment significantly increased C_p_ (from 39 to 52–56 °C). Interestingly, the Ph14 block copolymer did not exhibit thermoresponsive properties within the temperature range of 10–70 °C.

Previously it was shown that similar copolymers could form unimolecular micelles (or single chain nanoparticles, SCNPs) in water due to self-folding when they reached a certain degree of polymerization. Low-molecular weight copolymers of similar composition, which are unable to fold (due to limited flexibility), have to form multimolecular micelles to reduce the contact surface of hydrophobic units with water. This phenomenon has been studied in detail in a series of works [26,27,28,29,30].

Earlier it was demonstrated that the molecular weights determined by NMR and SLS methods in organic solvents agree well [37]. Therefore, the values of M_W_ obtained in acetonitrile were assumed to be true. Thus, the number of macromolecules in a micelle can be calculated as follows: N_agg_ = $M_{{W,H}_{2}O}$/$M_{W,\mathrm{ACN}}$. As can be seen from Table 2, the amphiphilic random copolymers obtained in this work were also capable of forming unimolecular micelles in aqueous solutions. The DLS data also confirmed the presence of narrowly dispersed monomodal particles with sizes comparable to the sizes of individual macromolecules in aqueous solutions. This was also evidenced by the positive and near-zero values of the second virial coefficients, A_2_. Comparing Rh for samples in water and acetonitrile indicated that all random copolymers formed sufficiently dense micelles in water; the exception was the hydrophilic MPEGMA homopolymer. Regarding the block copolymer, it formed huge multimolecular micelles in water with a diameter of ~320 nm, due to self-assembly.

## 4. Conclusions

Amphiphilic random and diblock thermoresponsive OEGMA-based bottlebrushes were successfully synthesized via photoiniferter RAFT polymerization. Copolymers with high DPs and reasonably good dispersities (1.2–1.3) were synthesized under cheap and safe household light sources at monomer conversion up to 85% reached in 75 min. The “on/off” photo-switchability of polymerization was demonstrated. The pseudo-living mechanism of polymerization and high chain-end fidelity were confirmed by successful chain extension.

The copolymer bottlebrushes showed LCST behavior, which could be finely tuned by varying the copolymer composition. In water, these copolymer bottlebrushes formed uni- and multimolecular micelles with narrow size distribution due to self-folding and self-assembly, depending on the copolymer molecular weight and architecture. DSC experiments revealed microphase separation in the block copolymer.

It was shown that GPC yielded inadequate values for OEGMA-based bottlebrushes in the high molecular weight region, due to the nonlinear dependence of the retention time on the molecular weight passing through the maximum.

## Figures and Tables

**Table 1:** Photoiniferter (co)polymerization of MPEGMA and AOEGMA.

ID	[MPEGMA]_0_/[AOEGMA]_0_/[CDTPA]_0_	Solvent	Light (Intensity, mW/cm^2^)	Time, min	Conversion, %	Composition ^a^, m_1_:m_2_ (mol)	M_n,th_^b^	M_n_^c^	M_W_^c^	PDI ^c^ (M_W_/M_n_)
Ph1	200:0:1	Toluene	green (7.3)	120	43	100:0	41,500	16,700	23,300	1.39
Ph2	500:0:1	Toluene	green (7.3)	180	48	100:0	121,000	15,700	26,200	1.66
Ph3	1000:0:1	Toluene	green (7.3)	210	47	100:0	242,400	22,200	36,700	1.64
Ph4	0:500:1	Toluene	green (7.3)	480	45	0:100	141,600	56,000	89,700	1.60
Ph5	200:0:1	DMSO	blue (7.0)	75	85	100:0	81,000	-	-	-
Ph6	200:0:1	Toluene	blue (7.0)	100	46	100:0	44,000	3800	4800	1.25
Ph7	200:0:1	THF	blue (7.0)	75	52	100:0	50,700	-	-	-
Ph8	200:0:1	Toluene	blue (8.3)	100	72	100:0	68,400	3600	4600	1.26
Ph9	200:0:1	Toluene	blue (8.3)	60	54	100:0	51,500	3400	4300	1.26
Ph10	100:100:1	Toluene	green (7.3)	480	65	51:49	74,000	40,800	56,200	1.27
Ph11-6 ^d^	100:100:1	Toluene	blue (8.3)	68	64	51:49	70,300	16,000	20,300	1.27
Ph12	100:100:1	DMSO	blue (7.0)	60	58	56:44	59,200	11,700	15,400	1.32
Ph13	0:200:1	THF	blue (8.3)	60	40	0:100	46,300	2900	3500	1.19
Ph14 ^e^	800:480:1	THF	blue (8.3)	90	42	42:58	390,000	40,400	49,700	1.23
R3 ^f^	80:80:1:0.2	Toluene	AIBN	360	83	52:48	22,800	16,300	19,800	1.22

^a^ Determined by ^1^H NMR and HPLC. For more details on ^1^H NMR and IR characterization of (co)polymers see Supplementary Materials. ^b^ Theoretical molecular weight calculated using the following equation: M_n,th_ = ([M_1_]_0_ × M_W_M_1_ × X_1_ + [M_2_]_0_ × M_W_M_2_ × X_2_)/[RAFT]_0_ + M_W_RAFT, where [M_1_]_0_, [M_2_]_0_, [RAFT]_0_, M_W_M_1_, M_W_M_2_, X_1_, X_2_, and M_W_RAFT correspond to initial concentrations of the monomers, RAFT agent, molar weights of the monomers, their conversions, and molar weight of RAFT agent. ^c^ Determined by GPC in THF with PSt standard calibration. ^d^ This line indicates the final polymer of the Ph11 series. During copolymerization, aliquots of the reaction mixture were taken after a specific time. The copolymers were isolated and used for further analyses on M_n,UV_ (Figure 7b) and thermoresponsive properties. ^e^ Ph14 is a chain extension from Ph13. Total monomer concentration 19%; in other experiments 50%. ^f^ R3 was obtained previously [31] through conventional RAFT polymerization in the presence of AIBN. The data are presented to evaluate the effect of side chain structure on thermoresponsive properties (see Section 3.3).

**Table 2:** Hydrodynamic and molecular weight characteristics of selected copolymers.

ID	Hydrodynamic Radius, R_h_, nm		M_n,UV_^a^	DP_n_^b^	${M_{W,\mathrm{ACN}}}^{c}$	$A_{2,\mathrm{ACN}}\;\times \;10^{4},$ mole cm^3^ g^−2^ ^c^	${M_{{W,H}_{2}O}}^{c}$	$A_{{2,H}_{2}O}\;\times \;10^{4},$ mole cm^3^ g^−2^ ^c^	N_agg_^d^
	**Acetonitrile**	**Water**							
Ph1	6.4	6.8	122,800	261	126,500	2.18	152,900	1.66	1.21
Ph10	8.4	5.9	199,400	194	221,900	0.54	221,500	0.43	~1.0
Ph12	7.3	5.8	126,100	136	214,300	0.41	225,700	−0.29	1.05
Ph14	-	162	480,900	378	-	-	-	-	-

^a^ Number average molecular weights determined by UV–visible spectroscopy in acetonitrile and methylene chloride. ^b^ Number average degree of polymerization calculated from M_n,UV_ and copolymer composition (Table 1). ^c^ Absolute weight average molecular weights and second virial coefficients determined by SLS in acetonitrile and water. ^d^ Aggregation number in H_2_O: N_agg_ = $M_{W,\mathrm{ACN}}$(SLS)/$M_{{W,H}_{2}O}$(SLS).

## Data Availability

The data presented in this study are available upon request from the corresponding author.

## Supplementary Materials

The following are available online at https://www.mdpi.com/article/10.3390/polym14010137/s1, Figure S1: Figure S1: ^1^H NMR spectrum of Ph4 homopolymer (pAOEGMA) in CDCl_3_, Figure S2: ^1^H NMR spectrum of Ph8 homopolymer (pMPEGMA) in DMSO-d6, Figure S3: IR spectrum of Ph8 homopolymer (pMPEGMA), Figure S4: ^1^H NMR spectrum of Ph10 comopolymer in DMSO-d6, Figure S5: ^1^H NMR spectrum of Ph11 comopolymer in DMSO-d6, Figure S6: ^1^H NMR spectrum of Ph12 comopolymer in DMSO-d6, Figure S7: IR spectrum of Ph12 comopolymer, Figure S8: ^1^H NMR spectrum of Ph14 block comopolymer in CDCl_3_, Figure S9: IR spectrum of Ph14 block comopolymer.

## Abbreviations

AOEGMA	alkoxy oligo(ethylene glycol) methacrylate
CDTPA	4-cyano-4-[(dodecylsulfanylthiocarbonyl)sulfanyl]pentanoic acid
C_p_	cloud point
CTA	chain transfer agent
DLS	dynamic light scattering
DSC	differential scanning calorimetry
LCST	lower critical solution temperature
LED	light-emitting diode
LMA	lauryl methacrylate
MA	methacrylic acid
MMA	methyl methacrylate
M_n,GPC_	number average molecular weight determined through gel permeation chromatography
M_n,th_	theoretical number average molecular weight
M_n,UV_	Number average molecular weight determined through UV–Vis spectroscopy
MPEGMA	methoxy oligo(ethylene glycol) methacrylate
OEGMA	oligo(ethylene glycol) methacrylate
PEG	polyethylene glycol
PEGMA	poly(ethylene glycol) methacrylate
PET-RAFT	photo-induced electron/energy transfer RAFT
RAFT	reversible addition−fragmentation chain-transfer
SCNP	single chain nanoparticle
SLS	static light scattering
