# Supplementary material for: Random and Diblock Thermoresponsive Oligo(ethylene glycol)-Based Copolymers Synthesized via Photo-Induced RAFT Polymerization
Source: Polymers (Basel). 2021 Dec 30;14(1):137. doi: 10.3390/polym14010137 (PMC8747269; doi:10.3390/polym14010137)
Supplement: Supplementary file 1 [file polymers-14-00137-s001.zip › polymers-1529889-supplementary.pdf]

Supplementary Information for:

Random and Diblock Thermoresponsive Oligo(ethylene glycol)-based Copolymers Synthesized via Photo-Induced RAFT Polymerization

Alexey P. Sivokhin[1,*], Dmitry V. Orekhov[1], Oleg A. Kazantsev[1], Olga S. Sivokhina[2], Sergey V. Orekhov[1], Denis M. Kamorin[1,3], Ksenia V. Otopkova[1], Michael A. Smirnov[1], Rostislav G. Karpov[1]

[1]Laboratory of Acrylic Monomers and Polymers, Department of Chemical and Food Technologies, Dzerzhinsk Polytechnic Institute, Nizhny Novgorod State Technical University n.a. R.E. Alekseev, 24 Minin Street, 603950 Nizhny Novgorod, Russia; sivokhin@dpingtu.ru (A.S.); mitriy07@mail.ru (D.O.); kazantsev@dpingtu.ru (O.K.); orekhov807@gmail.com (S.O.); d.kamorin@mail.ru (D.K.); k.otopkova@gmail.com (K.O.), thelordoftime@yandex.ru (M.S.);r_karpov@mail.ru (R.K.)

[2]V.A. Kargin Research Institute of Chemistry and Technology of Polymers with Pilot Plant, Dzerzhinsk, 606000, Russia, Dzerzhinsk 606000, Nizhegorodskaya obl., Russia; olgasivokhina@yandex.ru (O.S.)

[3]Chromatography Laboratory, Department of Production Control and Chromatography Methods, Lobachevsky State University of Nizhni Novgorod, Dzerzhinsk Branch, 23 Prospekt Gagarina, 603950 Nizhny Novgorod, Russia

* Correspondence: sivokhin@dpingtu.ru; Tel.: +7-831-334-7166

Synthesis of 4-cyano-4-(dodecylsulfanylthiocarbonyl)sulfanyl pentanoic acid

n-Dodecylthiol (15.4 g, 76 mmol) was added slowly to a stirred suspension of sodium hydride (3.15 g, 79 mmol) in diethyl ether (150 mL) at a temperature <10 ºC. The reaction mixture was cooled to 0 ºC, and carbon disulfide (6.0 g, 79 mmol) was added to give a yellow precipitate of sodium S-dodecyl trithiocarbonate separated by filtration.

To a suspension of sodium S-dodecyl trithiocarbonate (14.6 g, 0.049 mol) in diethyl ether (100 mL), iodine (6.3 g, 0.025 mol) was added. The reaction mixture was then stirred at room temperature for 1 h, and the precipitated white sodium iodide was removed by filtration. The yellow filtrate was washed with an aqueous solution of sodium thiosulfate to remove excess iodine and dried over sodium sulfate, and evaporated to give a residue of bis-(dodecyl-sulfanylthiocarbonyl) disulfide. A solution of 4,4'-azobis(4-cyanopentanoic acid) (2.10 g, 0.0075 mol) and bis-(dodecylsulfa-nylthiocarbonyl) disulfide (2.77 g, 0.005 mol) in ethyl acetate (50 mL) was heated at reflux for 18 h. After removal of the volatiles in vacuo, the crude product was extracted with water (5 ×100 mL) to obtain 4-cyano-4-(dodecylsulfanylthiocarbonyl)sulfanyl pentanoic acid as a pale yellow solid (3.65 g, 87% yield), mp 58–59 ºC, after recrystallization from hexane.

Characterization techniques

^{1}H NMR spectra were recorded at 25°C in $CDCl_3$ or DMSO-D6 on an Agilent 400 MHz DD2 spectrometer. FT-IR spectra were obtained from thin polymer films deposited on ZnSe glasses by evaporation of polymer solutions in diethyl ether (for all (co)polymers except for polyMPEGMA) and methylene chloride for polyMPEGMAs. Spectra were recorded on a Shimadzu IRAffinity-1 spectrometer with a resolution of 4 cm^{-1} and 20 accumulated scans.

The concentrations of the monomers in reaction mixtures were measured by HPLC using a Shimadzu Prominence chromatographic system equipped with a refractometer and matrix UV detector, a thermostat and a Kromasil 100-5-C18 4.6 x 250 mm column. Acetonitrile was used as an eluent, the flow rate was 0.9 ml/min, and the thermostat temperature was 55°C.

The compositions of copolymers at each conversion were calculated from the monomer consumption. At certain intervals, the current monomer concentrations were determined by HPLC; the copolymer composition (m_1) and the current conversion were calculated according to the following equations:

$$m_1 = \frac{M_1^0 - M_1}{(M_1^0 - M_1) + (M_2^0 - M_2)} \cdot 100\%,$$

$$Conversion = \frac{(M_1^0 - M_1) + (M_2^0 - M_2)}{M_1^0 + M_2^0} \cdot 100\% ,$$

where m_1 is the content of monomer 1 units in a copolymer, mol%; M_1^0 and M_2^0 are the initial concentrations of monomers 1 and 2 in the feed, mmol/g; M_1 and M_2 are the current concentrations of monomers 1 and 2, mmol/g.

The copolymer compositions determined by HPLC agreed well with those determined by ^{1}H NMR spectroscopy.

From ^{1}H NMR data (Figures S4-S6 and S8), the copolymer compositions were determined based on the ratio of the signal intensities of the protons corresponding to the methyl group (i) contained in MPEGMA units and the methylene group (c) contained in both MPEGMA and AOEGMA units. The compositions were calculated by solving the following equations:

$I_i=3N_{MPEGMA}$
$I_c=2N_{MPEGMA}+2N_{AOEGMA}$,

or

$I_i=3N_{MPEGMA}$
$I_d=32N_{MPEGMA}+22.8N_{AOEGMA}$,

where I_i, I_c and I_d are the signal integrals; N_{MPEGMA} and N_{AOEGMA} are the numbers of the MPEGMA and AOEGMA repeating units in a macromolecule.

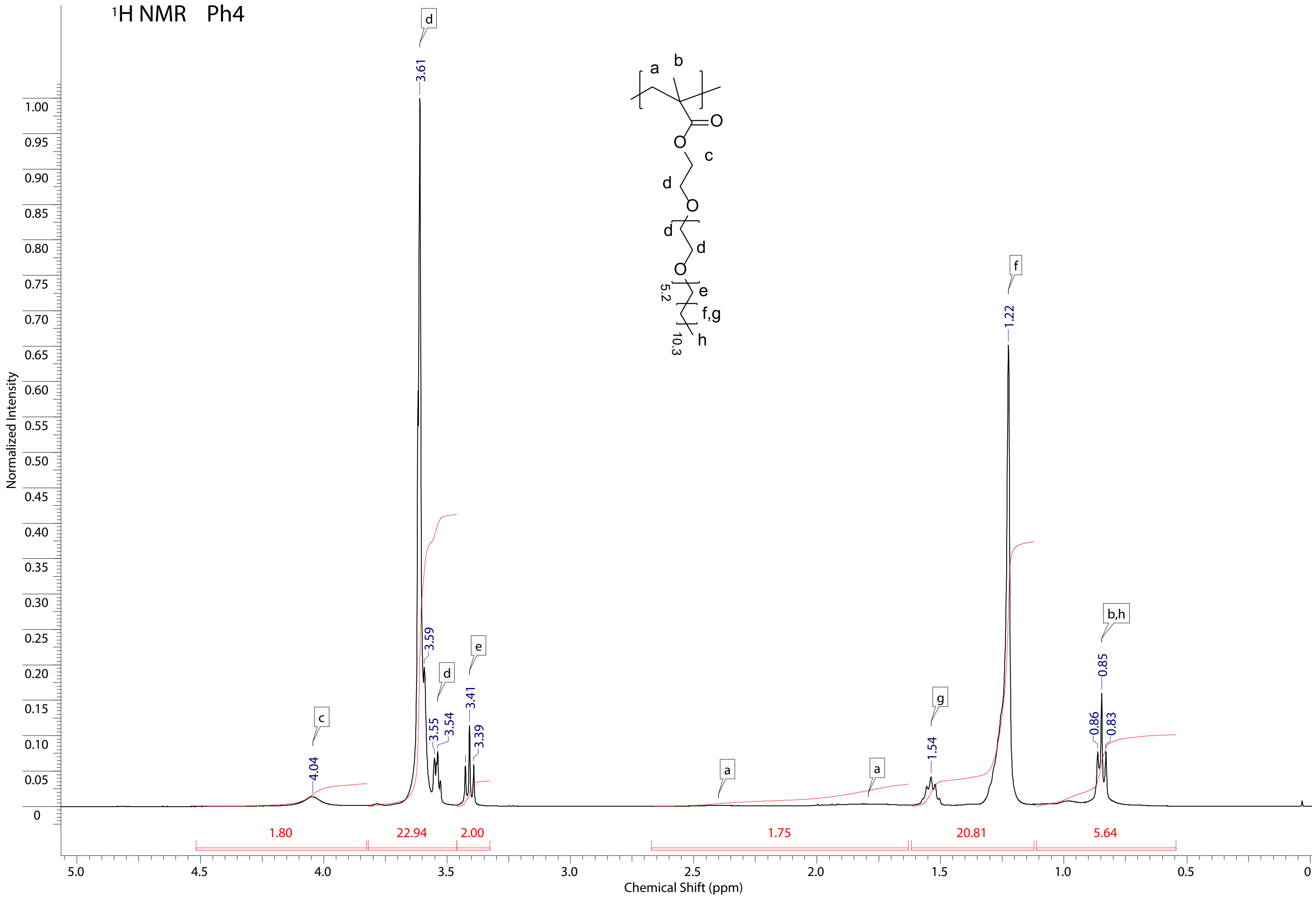

Figure S1. ^{1}H NMR spectrum of Ph4 homopolymer (pAOEGMA) in $CDCl_3$

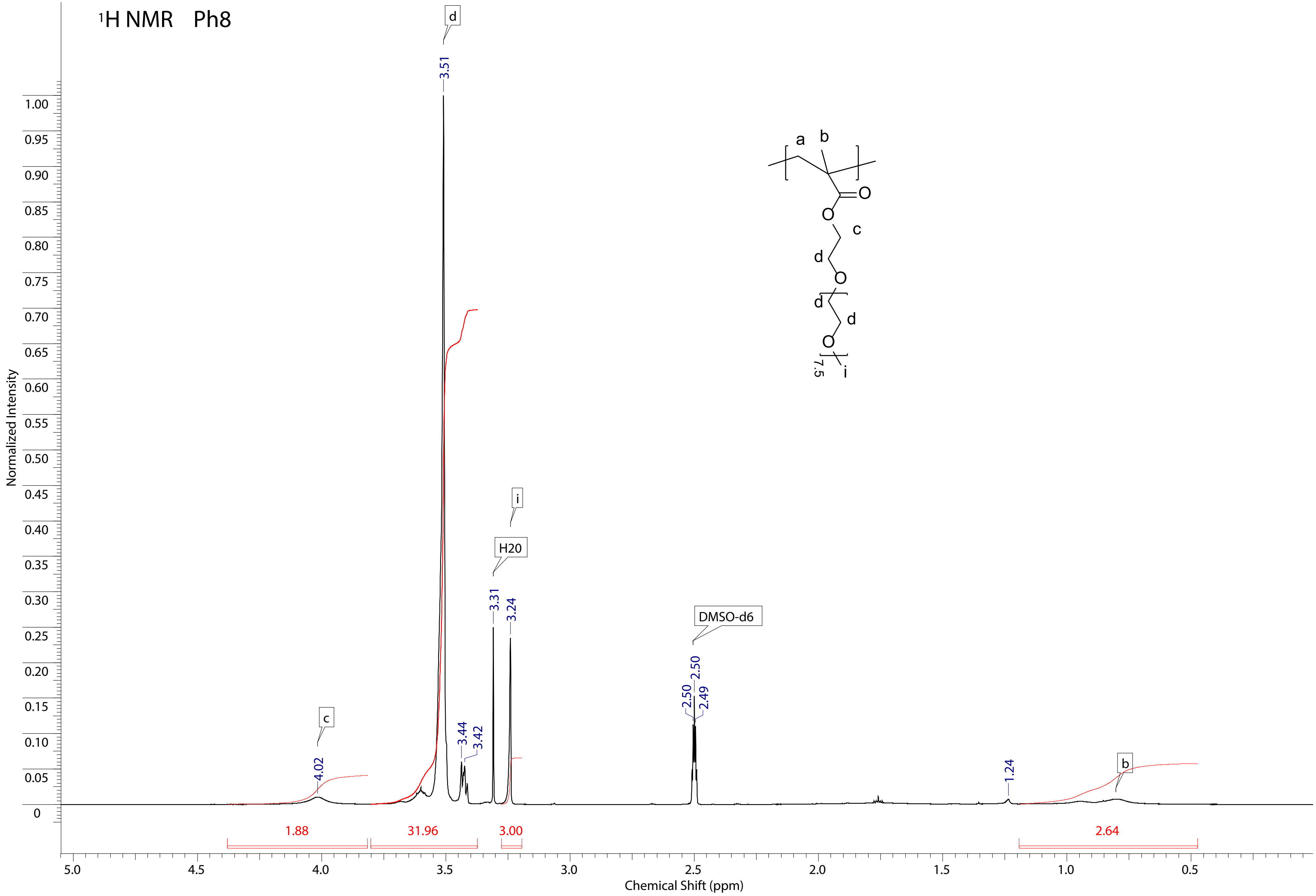

Figure S2. ^{1}H NMR spectrum of Ph8 homopolymer (pMPEGMA) in DMSO-d6

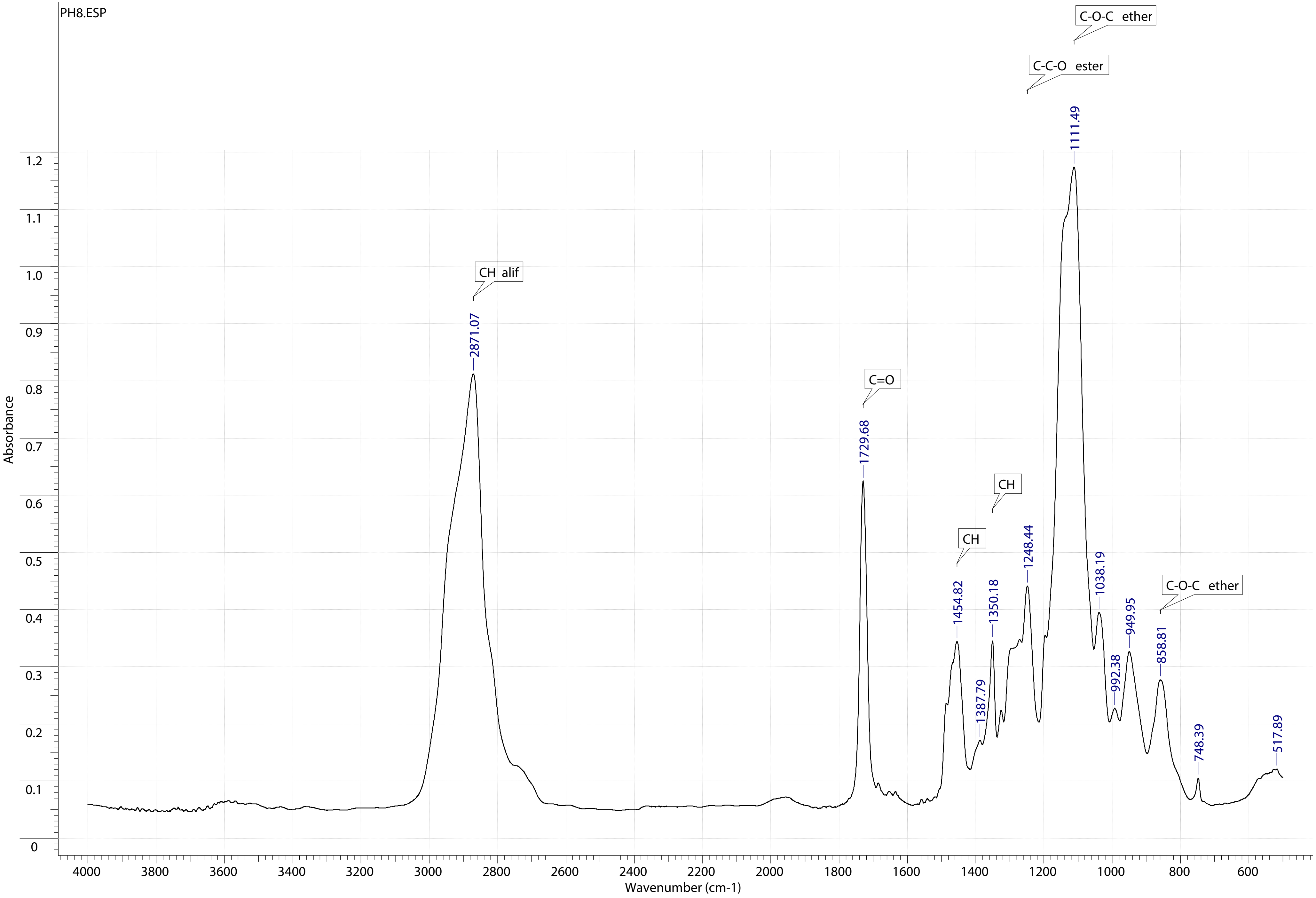

Figure S3. IR spectrum of Ph8 homopolymer (pMPEGMA)

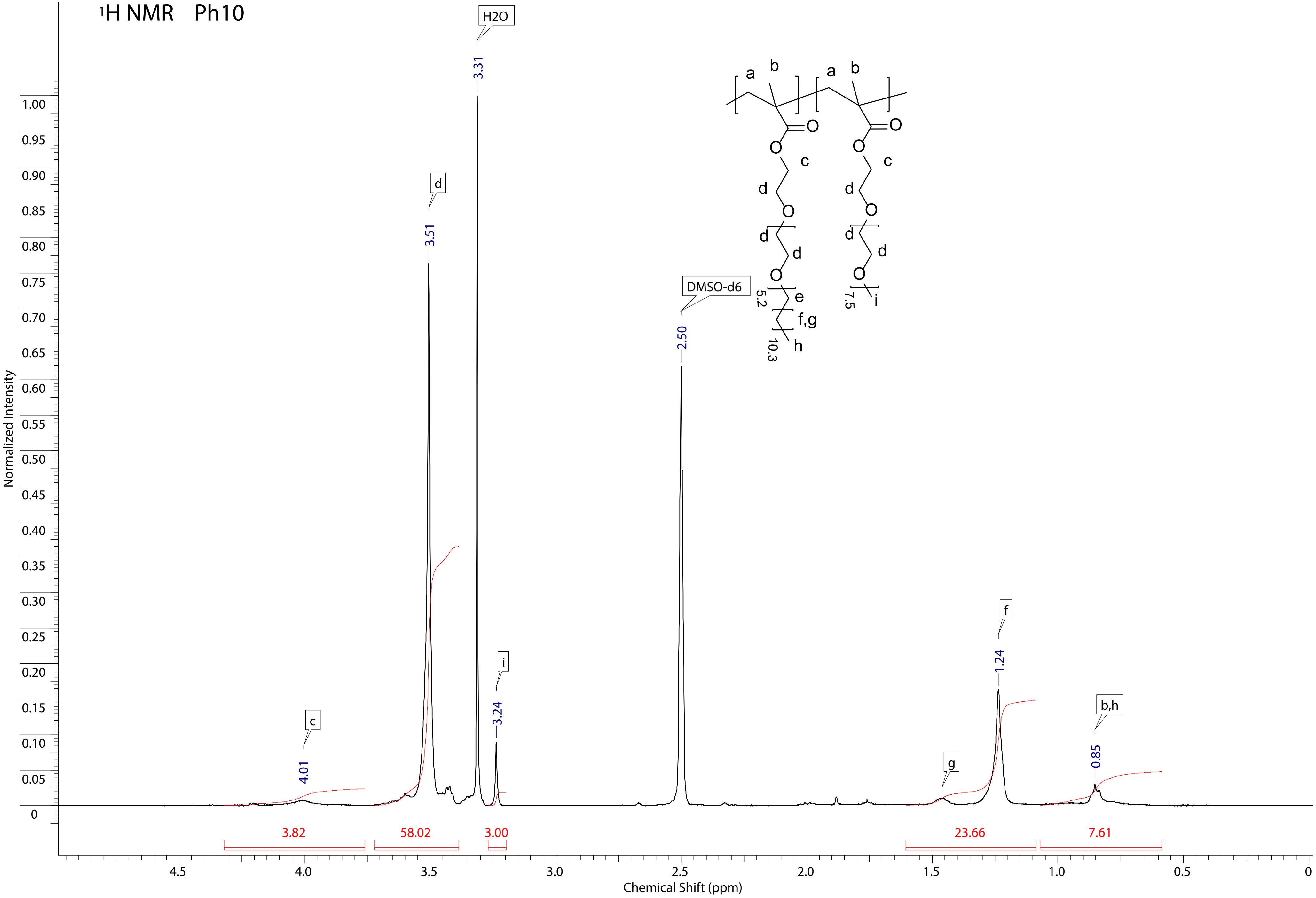

Figure S4. ^{1}H NMR spectrum of Ph10 comopolymer in DMSO-d6

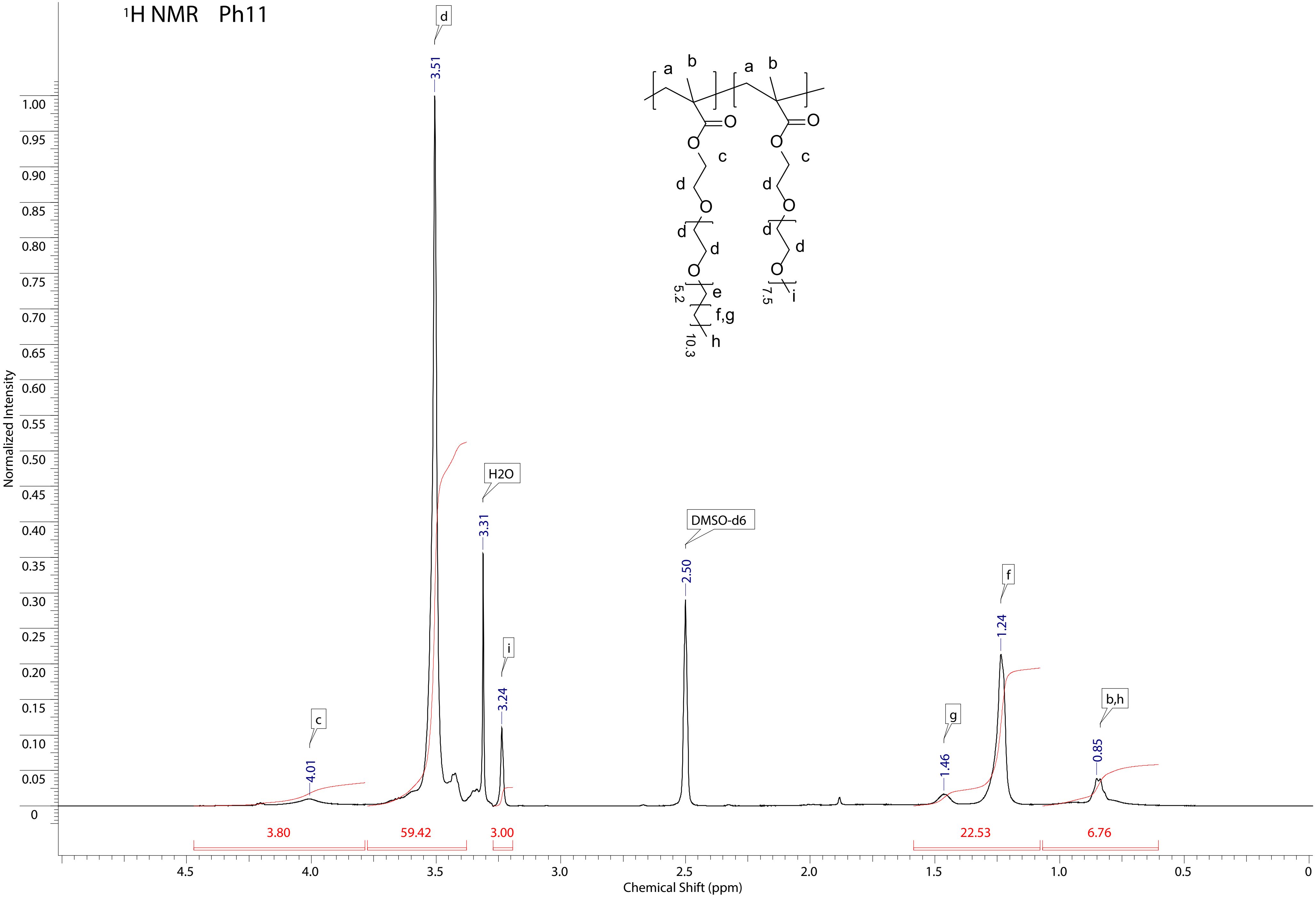

Figure S5. ^{1}H NMR spectrum of Ph11 comopolymer in DMSO-d6

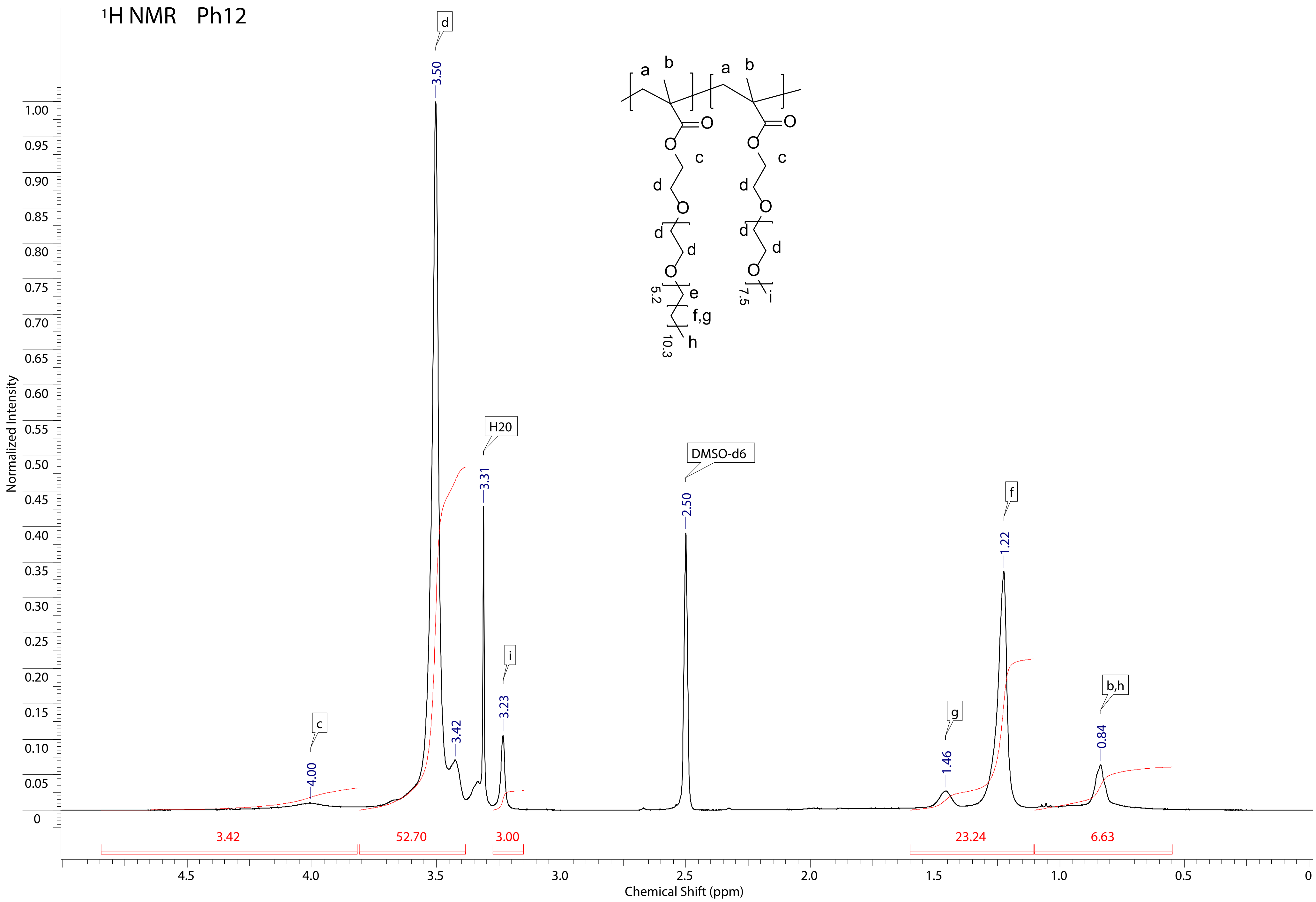

Figure S6. ¹H NMR spectrum of Ph12 comopolymer in DMSO-d6

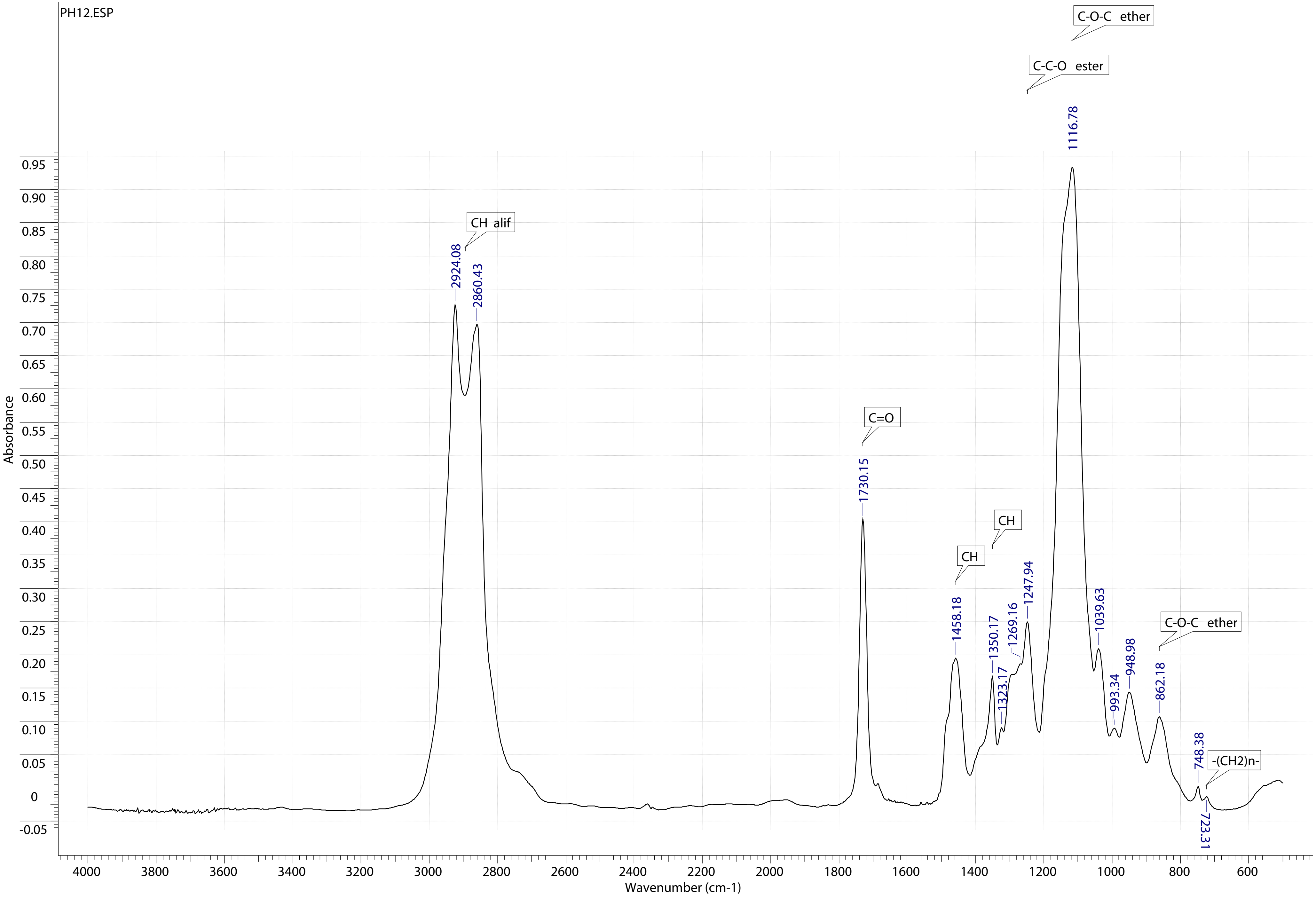

Figure S7. IR spectrum of Ph12 comopolymer

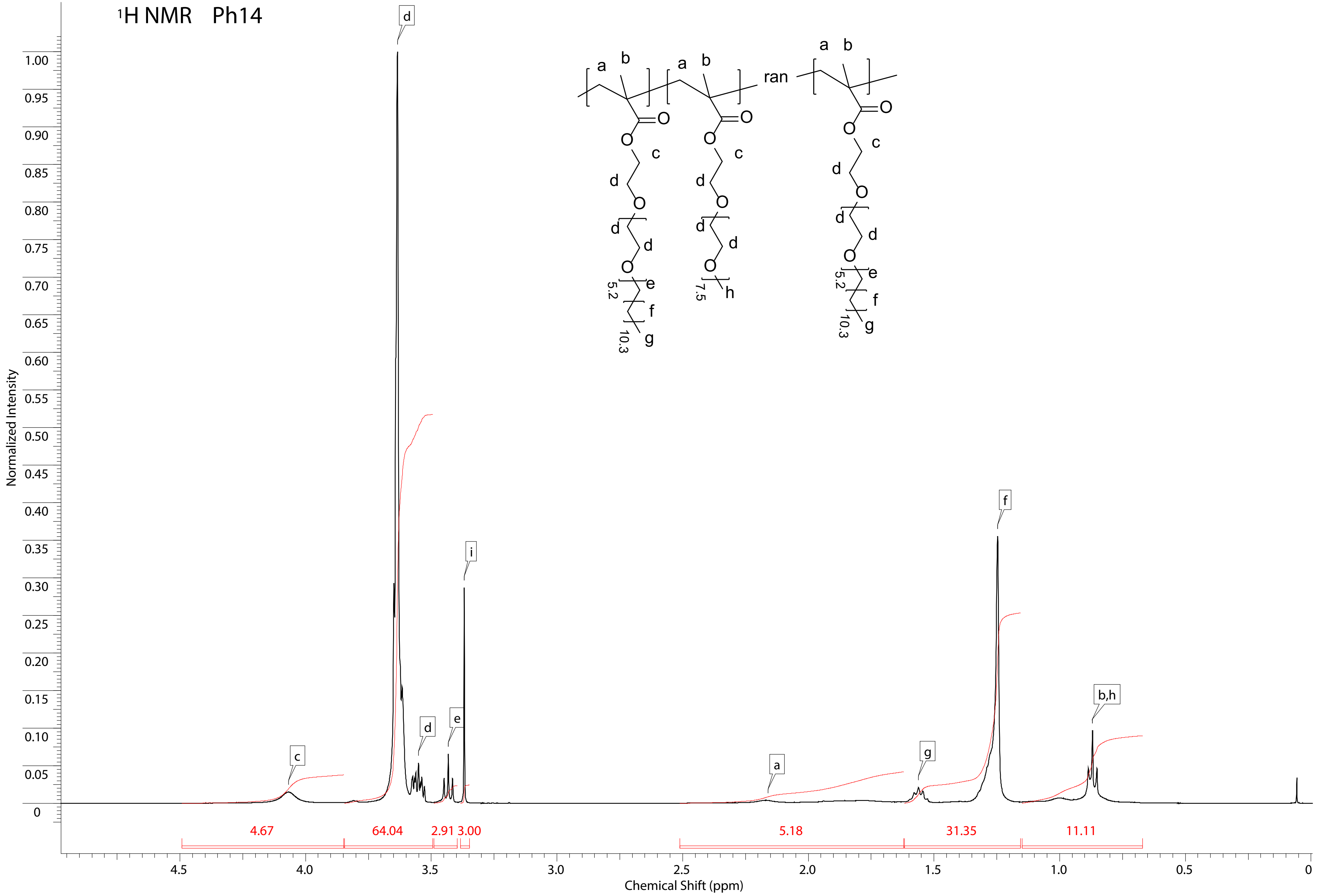

Figure S8. ^{1}H NMR spectrum of Ph14 block comopolymer in $CDCl_3$

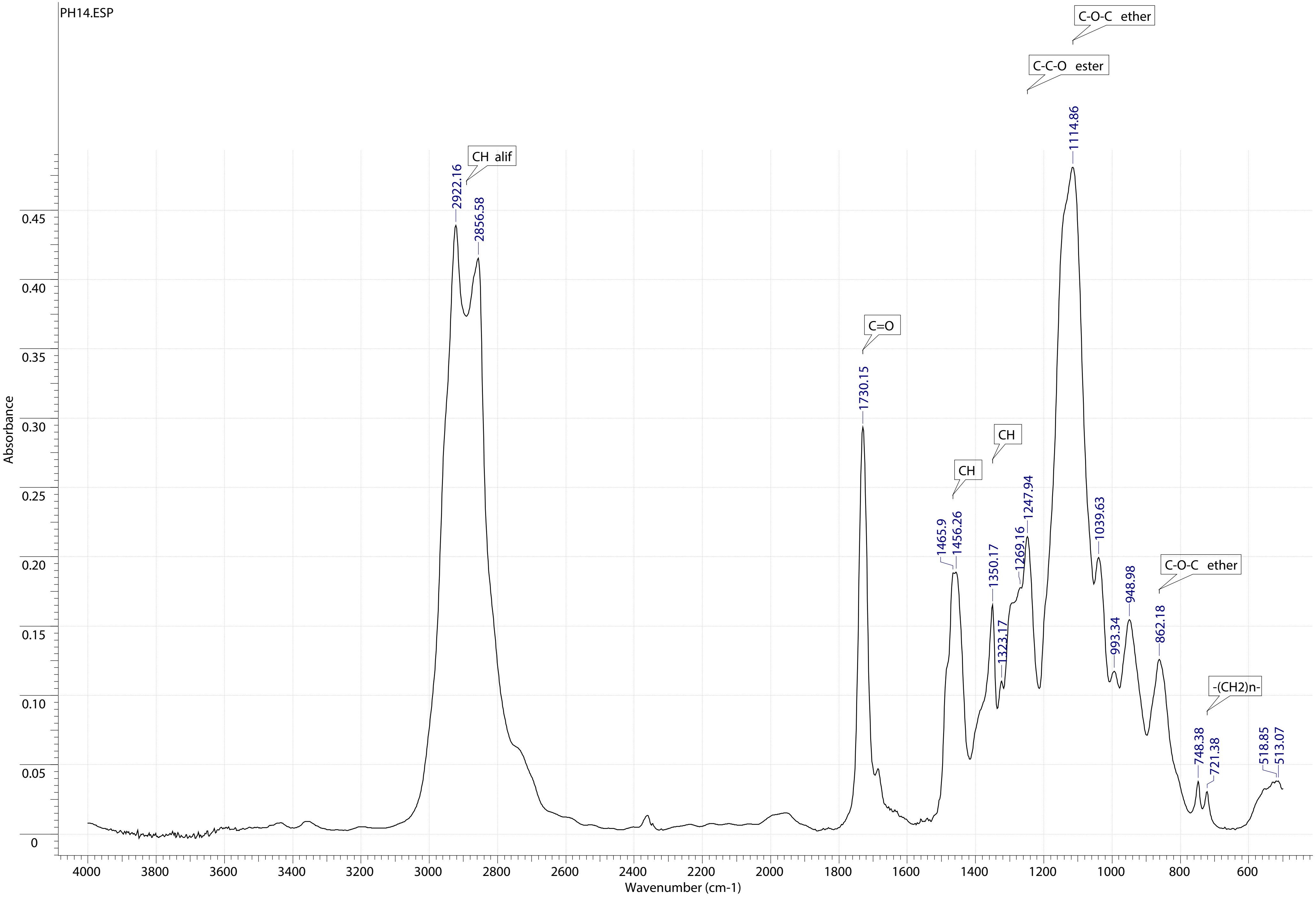

Figure S9. IR spectrum of Ph14 block comopolymer
